# Clinical and Histological Co-occurrence of Tropical Sprue and Helicobacter pylori Infection in Northeastern India: An Observational Study

**DOI:** 10.7759/cureus.87504

**Published:** 2025-07-08

**Authors:** Siddharth Shukla, Shashi Shekhar Prasad, Neha Mishra, Anupam K Singh, Chetan Sood, Shruti Vashisht

**Affiliations:** 1 Gastroenterology and Hepatology, Base Hospital, Guwahati, IND; 2 Pathology, Base Hospital, Guwahati, IND; 3 Dentistry, Base Hospital, Guwahati, IND; 4 Gastroenterology and Hepatology, Postgraduate Institute of Medical Education and Research, Chandigarh, IND; 5 Orthopedic Surgery, Armed Forces Medical College, Pune, IND; 6 Medical Education and Research Unit, Base Hospital, Guwahati, IND

**Keywords:** association, h. pylori, northeastern india, pathology, tropical sprue

## Abstract

Background: Tropical sprue (TS) and infection caused by *Helicobacter pylori* are a frequent cause of morbidity in the tropics. These disease entities, apart from having common regional distribution, probably also share hosts, sometimes exhibit symptom overlap, and very likely modify each other’s course. The data supporting this association is scant and may testify to the association between them in causing human suffering.

Methods: We enrolled 67 patients with dyspepsia, who also had an associated history suggestive of malabsorption (any two of the following: chronic diarrhea greater than four weeks and/or unintentional significant weight loss of >5% body weight over three months and/or nutritional deficiency of iron, B12, or folate in various proportions). They presented to the gastroenterology referral OPD of a tertiary care hospital in the northeastern region of India over a period of two and a half years. These patients were incorporated after informed consent and after fulfilling the inclusion and exclusion criteria. They initially underwent a battery of blood and stool tests to exclude other prevalent causes of malabsorption. This was followed by upper gastrointestinal endoscopy with additional rapid urease testing (RUT) wherever indicated, and mandatory biopsies were taken from the stomach corpus, antrum, and the second part of the duodenum.

Results: A significant 53 of 67 patients were found to have *H. pylori* infection (as diagnosed by endoscopic findings, RUT, and histopathological examination of gastric biopsies) and concurrent features of TS on duodenal biopsies.

Conclusion: Literature supporting such a close association between TS and *H. pylori* is scarce, and presentation of TS as dyspepsia is peculiar and unusual. This study offers new insights into the symptom overlap and coexistence of *H. pylori* infection and TS. In patients presenting with *H. pylori* infection and features of malabsorption, it is clinically prudent to evaluate for underlying TS to ensure a more inclusive and effective treatment approach.

## Introduction

Tropical sprue (TS), also known as tropical enteropathy, is a malabsorptive disorder endemic to many tropical and subtropical regions, particularly in South and Southeast Asia, the Caribbean, and parts of South America. It is a cause for significant morbidity in populations residing in tropical regions around the world and is challenging in both its diagnosis and management. A study by Ghoshal et al. in Lucknow, India, found that among 275 patients, 37% had TS [1]. TS usually presents as malabsorption (involving predominantly carbohydrate, vitamins, and minerals) and is defined by the presence of chronic diarrhea or significant unintentional weight loss, along with single or multiple nutritional deficiencies and abnormalities of the entire small bowel mucosa [2]. It is characterized by chronic inflammation and pathological alterations of the small intestinal mucosa, manifesting clinically as chronic diarrhea, unintentional weight loss, and multiple micronutrient deficiencies, notably those involving vitamin B12, folate, and iron. The precise etiology of TS remains elusive, though a chronic infectious or postinfectious mechanism has been widely postulated. Histologically, TS includes incomplete villous blunting in the duodenum, severe inflammation with villous blunting in the terminal part of the ileum, and a significant eosinophil infiltration in the lamina propria [3]. No organism has been implicated for TS as yet, and its features at times overlap with celiac disease (CD) [4], bacterial overgrowth, jejunitis, small intestinal lymphoma, protein-losing enteropathy, and drug-induced enteropathy [5-8]. TS in itself is a precipitator of small intestinal bacterial overgrowth (SIBO), which can provoke inflammatory responses and activate the endothelium in the portal venous system [9]. SIBO may thus be considered part of the spectrum of TS rather than a separate entity.

Another notorious infectious agent, *Helicobacter pylori,* jostles for hosts in the same geographic locations and socioeconomic subsets of the population as TS [10]. *H. pylori*, a Gram-negative bacterium that inhabits the gastric mucosa, shares many epidemiological risk factors with TS, including poor sanitation, contaminated water supplies, and crowded living conditions. *H. pylori* infects approximately 50% of the global population, and in resource-poor countries, it increases to 70%. Infected patients typically present with dyspepsia, epigastric pain, gastroesophageal reflux, and peptic ulcers. In some cases, these patients may also exhibit malabsorption, leading to diarrhea, significant weight loss, and deficiencies of iron, B12, or folate [11]. The alteration of gastric pH by *H. pylori* and its indirect effects, such as changes in gut immunity and microbiota, may lead to symptom overlaps with TS. Several factors, such as contaminated water and food, suboptimal sanitation, a warm and humid climate, and malnutrition, are common denominators. However, their concurrency is underreported. This clinical gap, characterized by the lack of a structured approach to evaluate for TS in patients already diagnosed with H. pylori who exhibit signs of malabsorption, has critical implications. If unrecognized, TS can lead to persistent symptoms, chronic nutritional deficiencies, and systemic complications [12]. Therefore, this study was undertaken to explore the frequency, co-occurrence, and histological interplay of TS and *H. pylori* in a tropical population, to determine whether TS should be actively evaluated in *H. pylori*-infected patients presenting with malabsorptive symptoms. We attempt to report overlapping symptoms and an uncanny frequency of association of TS and *H. pylori* observed in 53 of 67 patients who presented to us with the predominant symptom of dyspepsia.

## Materials and methods

This observational study was conducted in a tertiary care hospital. The study included the patients presenting to the gastroenterology OPD of a tertiary care hospital in the northeastern region of India with dyspepsia. Dyspepsia was defined by the ROME IV criteria (one or more of the following: bothersome postprandial fullness, bothersome early satiation, and bothersome epigastric pain or burning with no underlying structural disease, including upper gastrointestinal endoscopy, UGIE). The ROME IV further qualifies the criteria as continuous symptom duration of more than three months and symptom onset of more than six months [13].

For the purpose of the study, weight loss was defined as the unintentional loss of more than 5% weight over a period of 6-12 months [14]. Similarly, chronic diarrhea was defined as loose or watery stools three or more times a day for at least four weeks [15]. The gastroenterologist documented the symptoms during OPD visits before the upper gastrointestinal endoscopic examination.

Diagnosis of TS was based on the following exhaustive criteria: 1) epidemiological: the study population enrolled resided in the latitudes consistent with prevalence of TS; 2) clinical: apart from dyspepsia, the susceptible enrolled patients in our study presented with symptoms of chronic diarrhea, unintentional significant weight loss, and one or more nutritional deficiencies (iron, B12, and folate levels); 3) endoscopic: on upper gastrointestinal endoscopy, we encountered reduced duodenal fold height and numbers. Approximately one-fourth had grooving and scalloping of the folds. Immunoglobulin A tissue transglutaminase (IgA tTG) levels were negative; and 4) histopathology. The small bowel biopsies in TS/environmental enteropathy reveal a malabsorption pattern, which can be present in a variety of conditions like CD, TS, small intestinal bacterial overgrowth (SIBO), nongluten protein sensitivity, inflammatory bowel disease (IBD), and common variable immunodeficiency.

The patients consuming drugs for chronic diseases like type 2 diabetes mellitus, hypertension, coronary artery disease, and hypothyroidism were excluded from the study due to the probability of drug-related confounding. Additionally, we excluded patients with a history of multiple antibiotic exposures in the past six months for unrelated ailments, those on antitubercular therapy (owing to the antibacterial properties of antitubercular therapy), and patients on frequent nonsteroidal anti-inflammatory drugs or regular proton pump inhibitors (daily usage for more than 30 days). A total of 138 patients reported to the OPD, and after initial screening as per eligibility criteria, 67 of them were included in the study. A written information sheet was provided to all patients in the local language. Thereafter, informed consent was obtained, and ethical clearance was obtained from the institutional ethics committee.

For the 67 recruited patients, complete blood count, corrected reticulocyte count, absolute eosinophil count, liver function tests including albumin and globin levels, renal function tests, serum iron studies(including serum iron, serum ferritin, transferrin saturation, and total iron binding capacity), corrected calcium levels, serum B12 and folate levels, quantitative C-reactive protein, IgA tTG, thyroid function testes hemoglobin A1c, stool routine and microscopic examination (RE/ME), stool-modified Ziehl-Neelsen (ZN) stain, stool occult blood (on 03 occasions), fecal calprotectin levels, and rapid test for HIV were conducted as per the protocol. An ultrasound of the abdomen was performed by a qualified radiologist to exclude gallstone disease or gross pancreatic pathology. Standardized lab cut-off values were taken. This battery of tests was employed to establish the predominant type of malabsorption and grossly exclude any underlying infective or other inflammatory conditions.

Then, the 67 participants underwent UGIE using high-definition white light endoscopy. Testing for *H. pylori* infection and TS was done by visual assessment on UGIE with/without rapid urease testing (RUT) and mandatory biopsies from the stomach (corpus and antrum) and duodenum. Primary identification of Helicobacter infection was made by suggestive UGIE findings and a positive RUT at 10 minutes. Confirmation was done by examination of biopsies obtained from the gastric corpus and antrum and staining them with hematoxylin and eosin (H&E), followed by Giemsa and examination under high power. Similarly, TS was diagnosed by clinical features of malabsorption and UGIE findings of duodenal mucosal atrophy on gross endoscopic examination (reduced duodenal fold height and numbers with occasional grooving and scalloping). Confirmation of the diagnosis was done by detailed histopathological examination of the biopsy of D2. We wish to emphasize the clinical importance of the Sydney system of classification of chronic gastritis here, and the same was applied in our biopsies to determine *H. pylori* density, degree of activity, chronic inflammation, atrophy, and intestinal metaplasia [16]. All patients had complete data for the required parameters.

## Results

Out of 67 patients with dyspepsia and malabsorption, 53, which will henceforth be referred to as cases, were detected to have features of both TS and *H. pylori* infection. The demographic characteristics and findings are as follows: 35 (66%) were men and 18 (34%) were women, with ages spanning from 22 to 76 years. Forty-nine cases took a mixed diet, including eggs, while four were vegetarians. None of them followed a vegan diet. Fifty of them were aboriginals, and three were permanent settlers from other parts of the country. All belonged to low to middle socioeconomic strata. Educational qualifications varied from unread to postgraduates. Five were smokers, and 14 occasionally consumed alcohol (all men and consumed a maximum of 10 cigarettes/day and 60-80 g twice to thrice a week).

**Figure 1:**
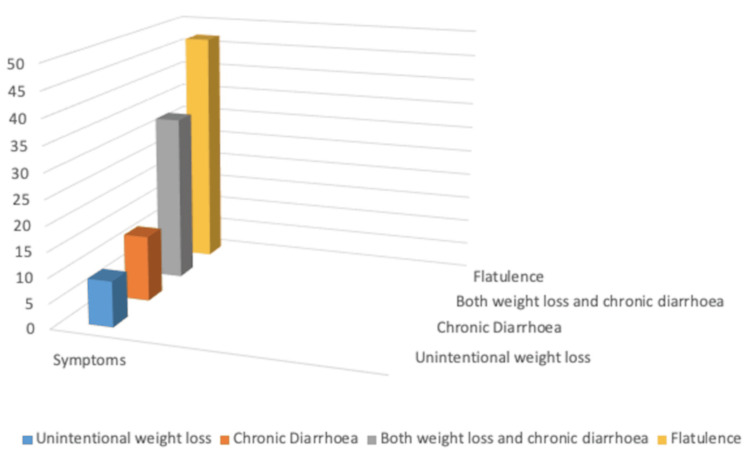
Proportion of various symptoms reported among 53 cases

All 53 cases presented with one of the following clinical manifestations of dyspepsia (each qualifying as per ROME IV): epigastric discomfort, bloating, early satiety, and heartburn. Malabsorption manifested as isolated chronic diarrhea in 11 (21%), isolated unintentional weight loss in nine (17%), and as both in 33 (62%) cases. It was primarily of carbohydrates, iron, B12, and folates. Forty-seven cases (88%) had additional complaints of flatulence. This was reflective of incompletely digested carbohydrates being fermented in the small intestinal lumen by the prevailing microbiota. The graph of symptoms is shown in Figure 1.

All cases demonstrated the presence of at least one nutritional deficiency (iron, B12, or folate). However, no characteristic dietary associations or habits were reported, nor did any case have a history suggestive of steatorrhea. Reported weight loss ranged from approximately 6% to up to 20% while symptoms of diarrhea ranged from seven weeks to up to one and a half years.

Endoscopic aspect

**Figure 2:**
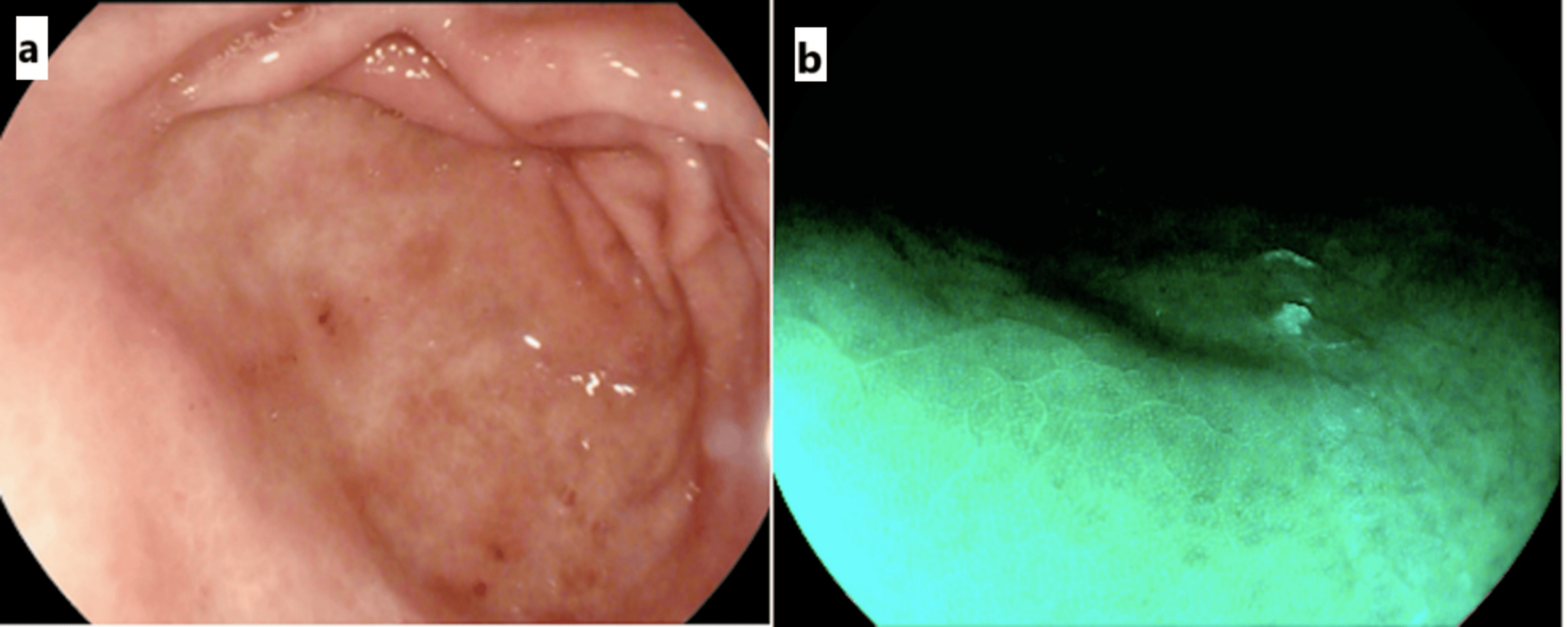
(a) H. pylori-associated antral predominant gastritis on UGIE. (b) An image enhanced endoscopic picture of antral gastric mucosa showing mosaic pattern suggestive of H. pylori infection UGIE: upper gastrointestinal endoscopy

UGIE showed features of antral predominant gastritis in 31 (58%), corpus predominant gastritis in six (11%), and pangastritis in 16 (30%) cases. Nodular gastric mucosa was also noted in five individuals (9.4%) (Figures 2a, 2b).

**Figure 3:**
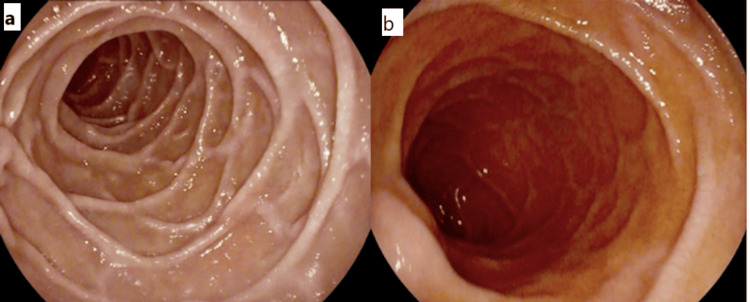
(a) Duodenal mucosa in its second part showing reduced fold height and numbers in an affected patient. (b) More pronounced changes in another patient with a near featureless duodenum

A notable reduction in both the height and number of folds in the duodenum was documented in all cases that underwent endoscopy. Further, grooving and/or scalloping of duodenal folds was identified in 13 cases (24%) (Figures 3a, 3b). A duodenal ulcer (in its first part) exhibiting signs of healing was noted in one patient. It was classified according to the Forrest Classification as grade III [17].

Histological aspect

**Figure 4:**
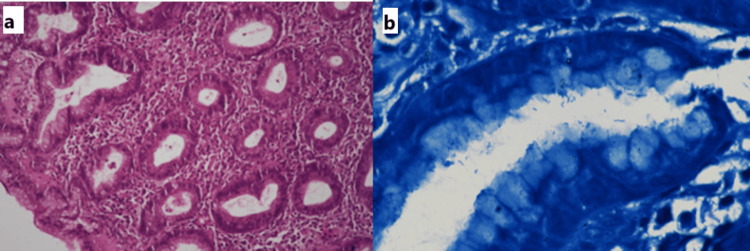
(a) H. pylori-associated gastritis. Note the marked mixed inflammation in the lamina propria with cryptitis and mucosal atrophy (H&E, 200x). (b) H. pylori clusters seen at higher resolution (Giemsa 1,000x) H&E: hematoxylin and eosin

Histologically, the spectrum of *H. pylori* infection and associated gastritis ranged from mild to severe density of activity, chronic inflammation, and atrophy. Intestinal metaplasia was not noted in any of the cases (Figures 4a, 4b).

**Figure 5:**
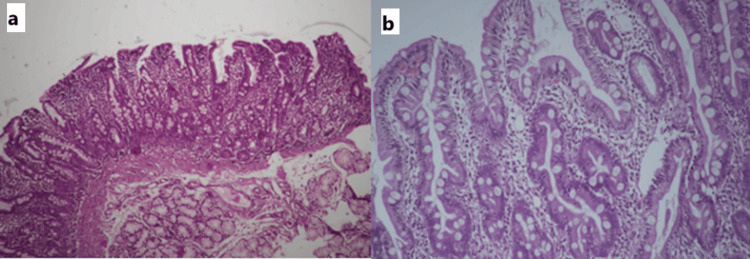
Tropical sprue biopsy from the second part of the duodenum from two different cases, showing (a) a variable degree of villous blunting ranging from a mild to a moderate degree (H&E, 100x) and (b) variable villous blunting with intraepithelial lymphocytosis more prevalent toward the crypts than the surface epithelium. Lamina propria is expanded with lymphomononuclear cells (H&E, 200x) H&E: hematoxylin and eosin

Duodenal biopsies revealed varying degrees of villous blunting and crypt hyperplasia with lamina propria infiltrated by lymphocytes, plasma cells, and eosinophils. IELs were more toward the crypts compared to surface epithelial cells. We also noted the presence of duodenal IELs characterized by mild to moderate villous blunting and expansion of the lamina propria due to lymphocytes, plasma cells, and eosinophils (Figures 5a, 5b). None of the biopsy samples indicated the presence of H. pylori in the duodenum.

## Discussion

TS, also known as environmental enteropathy, is an intestinal malabsorptive disorder affecting the entire small bowel, extending up to the ileum. It is diagnosed in an appropriate climatic, geographic, and patient’s socioeconomic condition when they present with characteristic symptoms of malabsorption. UGIE findings and typical histology of the affected duodenal mucosa help establish the diagnosis of TS [18]. Hillary first described such an illness in 1759 [19]. As the name implies, it affects the population residing between the two tropics. It may, therefore, be defined as a disease complex encompassing specific small bowel mucosal abnormalities leading to symptoms of malabsorption and nutritional deficiencies. Malabsorption in TS predominantly affects carbohydrates, vitamins, and minerals, explaining the consequent manifestations [20].

In their study, Ghoshal et al. suggested that one cause of malabsorption is contamination of the small bowel with aerobic bacteria [20]. This may lead to prolongation of orocecal transit time following activation of the ileal brake and a possible progression to TS. It suggests an infectious etiology of TS, which subsequently shows a good response to a prolonged course of tetracyclines and folic acid. Despite several attempts, no bacterial agents have been isolated yet [21]. A reason for the response to tetracyclines and folic acid could be the inherent anti-inflammatory activity of these two molecules, which may contribute to reepithelialization and improvement in gut functionality. Another etiology suggested is of a bacterial "hit and run," damaging enterocytes and causing subsequent effects. This likely initiates an event cascade involving small bowel stasis, bacterial overgrowth, resulting in malabsorption and folate deficiency, which further damages enterocytes, causing a vicious cycle [22]. Understandably, all these divergent data add to an air of ambiguity about the causality. Thus, treatment options in TS remain incompletely understood, while the disease has far-reaching consequences on several organ systems due to malabsorption and related nutritional deficiencies.

We assert that a comprehensive assessment of sprue-like illness is incomplete without a credible exclusion of CD, given the well-documented clinical and histological overlaps between CD and TS. CD is an immune-mediated enteropathy triggered by dietary gluten in genetically susceptible individuals and is characterized by malabsorption, chronic diarrhea, and nutritional deficiencies, symptoms that closely mirror those of TS. It is characterized under suitable clinical and demographic circumstances with raised serum antitissue transglutaminase antibody levels. On histopathology, CD has tip-heavy IELs with more pronounced villous blunting leading to flat mucosa and a histological response to a gluten-free diet. The probability of CD in our study population was low due to the geography and dietary preferences. Yet, an attempt was made to convincingly exclude it with IgA tTG levels and histology.

*H. pylori* presents as dyspepsia, epigastric or abdominal pain, and gastroesophageal reflux disease, as well as peptic ulcer disease and vitamin B12 deficiency. Histologically, it shows gastritis, gastric mucosa-associated lymphoid tissue lymphoma, and gastric adenocarcinoma [23]. In common with sprue-like illnesses, it often leads to duodenal IELs that mimic CD histologically [24]. However, small intestine biopsy in cases of *H. pylori* gastritis shows fewer IELs than CD, and villous blunting is almost never present. This attribute was taken into consideration while analyzing the duodenal biopsies of our patients. Underlying chronic diarrhea and nutritional deficiency anemia have also been reported with *H. pylori*. However, there is no significant villous atrophy in such cases [5,25]. This important finding helped us differentiate duodenal changes owing to *H. pylori* vs. those observed in TS.

In one study, Durán et al. reported that *H. pylori* infection causes gastric mucosal atrophy, resulting in reduced acid production. This phenomenon adversely affects the bactericidal ability of the stomach and changes the flora in the stomach and small intestine [25], which may trigger TS-like illness. In addition, *H. pylori* significantly diminishes the Firmicutes to Bacteroidetes ratio [26], and eradication of *H. pylori* increases helpful Bifidobacteria in the intestinal flora [27]. Furthermore, *H. pylori* and small intestinal bacterial overgrowth (SIBO) have been reported to coexist [28].

Sometimes various other malabsorptive entities like peptic duodenitis, medications, small intestinal bacterial overgrowth (SIBO), nongluten protein sensitivity, IBD, CVID, autoimmune enteropathy, and reactive duodenopathy may act as confounders too [29]. These conditions can be fairly ruled out based on clinical features, including population predisposition, history, serology, stool RE/ME, including ova cyst examination, stool modified ZN stain examination, fecal calprotectin, UGIE, and biopsy. We too made a sincere effort to exclude these variables.

Association between TS-like illness and *H. pylori* is not well established yet, and literature supporting such association is extremely limited, with a singular case reported by Vasudevan et al. from southern India, where *H. pylori* infection presented as chronic diarrhea and hypoalbuminemia. The UGIE was surprisingly normal in this case [11]. A clinical, endoscopic, and histopathological association of TS with *H. pylori* is hitherto unreported in available literature, especially for TS presenting as predominant dyspepsia. While a few more authors have noted changes in duodenal mucosa in patients infected with *H. pylori* in randomly selected samples, their studies predominantly highlighted a pathological perspective only [5,6,22,30].

Our study aims to highlight the notable differences in TS symptomatology and suggest the coexistence and interplay of TS with *H. pylori*. We have tried to systematically document the concurrent clinical, endoscopic, and histopathological features of TS and *H. pylori* infection in a defined regional population. While isolated reports have previously hinted at *H. pylori* presenting with atypical gastrointestinal symptoms such as chronic diarrhea or nutrient deficiencies, none have convincingly established its coexistence with TS in a diagnostically rigorous, cohort-based manner. Our findings reveal an unexpectedly high rate of dual pathology in patients presenting with predominant symptoms of dyspepsia and malabsorption, traditionally attributed to *H. pylori* alone, highlighting a need to reconsider prevailing diagnostic algorithms.

This observation suggests that in populations residing in or originating from tropical regions, especially where nutritional deficiencies and poor sanitation are endemic, a TS workup may be clinically warranted in patients diagnosed with *H. pylori* infection who also exhibit signs of malabsorption (e.g., unexplained weight loss, chronic diarrhea, iron, or B12 deficiency). Given the overlapping histopathological features and the subtlety of TS endoscopic findings, reliance solely on *H. pylori* status may lead to underdiagnosis of TS and suboptimal treatment outcomes. Therefore, our study advocates for a more integrative diagnostic approach in such patients, one that includes targeted small bowel biopsies and nutrient panels, to uncover underlying or concomitant TS. Further research will tell whether this relation was causal or incidental. With the possibility that *H. pylori* exacerbates TS through direct effects, this case series also proposes the need to consider them in conjunction and modify evaluation or treatment protocols accordingly.

As we conclude, this study is small and has several limitations. First, it is a single-center, unblinded study with a limited sample size. It is region-specific, with its own social and gastronomic uniqueness. UGIE and targeted biopsies were critical interventions with a fair degree of objectivity; yet, despite best efforts, they are vulnerable to observer biases. Other tests for confirming the causes of malabsorption, including fecal elastase for chronic pancreatitis, *H. pylori* virulence factors (CagA or VacA) and their effects, microbiota assessment, or the level of gastric acid secretion and consequent alterations in microbiota, could not be performed due to technical and financial limitations. Second, the diagnosis of TS is clinicopathological, and there are no gold standards available. So, qualifying patients as TS may have been erroneous at times. Third, TS and *H. pylori* are also known to affect the small intestine in its entirety, and a colonoscopy with terminal ileal evaluation, while desirable, was not part of this study. Additionally, there was no comparator or control group, as this was an observational study. Randomized, multicenter, and blinded studies in this regard are recommended, which may also provide a model for studying other intestinal disorders linked to infections or dysbiosis.

## Conclusions

This study aims to investigate the interplay between symptoms and the pathophysiology of *H. pylori* infection in patients with TS in a relatively remote tropical region of India. This study highlights a novel and clinically significant observation: the frequent co-occurrence of TS and *H. pylori* infection in patients presenting with predominant symptoms of dyspepsia and malabsorption in a tropical, resource-limited setting. Histopathological differentiation between TS, CD, and *H. pylori*-associated enteropathy remains a critical challenge. We addressed this by methodically excluding CD through serological and histological criteria, and by evaluating duodenal biopsy features to distinguish *H. pylori*-induced changes from those of TS.

Through this study, we aim to provide new insights into the overlapping presentation and interlinked pathophysiology of these two diseases. This may have diagnostic and possibly therapeutic implications. Despite limitations, this study can serve as a precursor for further clinical research with an emphasis on upper gastro intestinal infections, inflammation and microbial dysbiosis. This may further help us improve the current understanding of TS and suggest new diagnostic and therapeutic insights.
